# Editorial: Global Change, Clonal Growth, and Biological Invasions by Plants

**DOI:** 10.3389/fpls.2016.01467

**Published:** 2016-09-29

**Authors:** Fei-Hai Yu, Sergio R. Roiloa, Peter Alpert

**Affiliations:** ^1^School of Nature Conservation, Beijing Forestry UniversityBeijing, China; ^2^BioCost Group, Department of Animal Biology, Plant Biology and Ecology, Faculty of Science, University of A CoruñaSpain; ^3^Biology Department, University of MassachusettsAmherst, MA, USA

**Keywords:** climate change, clonality, environmental heterogeneity, genotypic and phenotypic diversity, invasiveness, rapid evolution

Global changes in climate, land use, nutrient availability, acidity, populations of harvested or undesired species, and concentrations of toxins are now widely evident. Their ecological and evolutionary consequences are likely to be great but are often hard to identify or anticipate because of the multiple interactions that shape most ecological systems. One potentially important set of interactions involves the properties of clonal growth in plants. Clonal growth is common in plants and in ecosystems around the world and appears to be associated with the invasiveness of introduced plant species. Emergent clonal traits such as resource sharing and signaling between connected plants within clones, selective positioning of plants during clonal growth, stores of energy or nutrients that can be reallocated between connected plants, meristem banks that can be initiated in response to clonal integration, and tradeoffs between clonal and sexual reproduction might contribute to plant invasiveness and community invasibility, and global change may affect the expression, fitness effects, and evolution of these traits. This research topic assembles articles that deal explicitly with interactions between clonal growth and plant invasion or global change, and additional papers that advance understanding of aspects of clonal growth likely to affect invasion or response to global change.

Four papers consider the evolution of clonality in plants. Epigenetic variation may provide an alternative means of adaptation to changing environments and play a key role during the invasion of some introduced clonal plants that cannot successfully reproduce sexually in the invaded range. In a forum paper, Dodd and Douhovnikoff propose how epigenetic variation may potentially enable clonal plants to adjust to changes in means and extremes of climate. Castro et al. report that plants of *Oxalis pes-caprae* from the native range show higher sexual fitness, while those from the invasive range have higher asexual fitness, suggesting that evolution has favored asexual over sexual reproduction during invasion. Roiloa et al. compare a different suite of clonal growth properties in *Carpobrotus edulis* from the native range in South Africa and the invaded range in Spain and Portugal. Plants from different ranges did not differ in degree of division of labor (i.e., specialization to acquire and share locally abundant resources), but the beneficial effect of clonal integration on the dry mass of apical ramets was higher in the introduced populations, suggesting that this clonal trait may have been selected for in the invaded range. Holmes et al. suggest that local adaptation in response to salinity has enabled clones of *Phragmites australis* from highly saline areas to function better under saline conditions in Australia. Their examination of gene expression using transcriptomics found clear differences in responses to salinity between clones sampled from areas with low and high salinity.

Reliance on asexual reproduction has generally been seen as a likely constraint on evolution in clonal organisms. Two papers shed new light on the question of whether asexual and sexual reproduction conflict. Huber et al. examine the implications of intraspecific variation in the size of ramets in *Trifolium repens* and find evidence for a tradeoff between net production of biomass and rate of vegetative reproduction. Evolutionary shifts in the genetic composition of clonal populations may thus depend on whether ramet size or ramet number is under stronger selection. The picture may be quite different at higher taxonomic levels. Xie et al. compile data on 115 species from 139 publications and find no consistent tradeoff between allocation to clonal organs and to sexual reproduction.

Three papers focus on the intensively studied, highly invasive, amphibious, clonal herb *Alternanthera philoxeroides*, which has been introduced from South America to Asia, Australia, and North America. *A. philoxeroides* shows lower genetic diversity and phenotypic plasticity in China than in Argentina or the USA (Geng et al.). Since relatively high plasticity has often been found to be associated with invasiveness, this may mean than capacity for clonal growth can to some extent substitute for plasticity in invasions by plants. You et al. simulate invasion by *A. philoxeroides* into both open areas and stands of a native wetland clonal plant, *Jussiaea repens*; propagule pressure and connection between plants both facilitate invasion, supporting a role for physiological integration in the invasiveness of clonal plants. Zhang et al. caution that this may depend upon nutrient availability: connection between ramets within pairs in *A. philoxeroides* decreased their combined growth in mass when the level of nutrients was high and decreased the production of new ramets when the level of nutrients was low.

Three papers introduce novel concepts or properties of clonal plants. Martínková and Klimešová present the concept of enforced clonality, vegetative reproduction due to damage in normally non-clonal plants. Root fragmentation increased fitness in *Barbarea vulgaris* but not *B. stricta*, suggesting that enforced clonality may shift the relative fitness of species of some non-clonal plants in frequently disturbed habitats. Waters and Watson provide evidence for clonal foraging in response to volatiles released from soil. Ramets of *Fragaria vesca* produced more root mass when exposed to air above non-sterilized soils than when exposed to air above sterilized soils, and stolons tended to grow toward non-sterilized soils rather than sterilized soils. This could provide a mechanism for detection of soil resource levels prior to rooting. In what may be the first study of the potential role of endophytes in invasion by clonal plants, Dai et al. show that infection by the endophyte *Bacillus* sp. promotes the growth of the invasive clonal plant *Wedelia trilobata* but not that of the congeneric, native, clonal plant *W. chinensis*.

One of the best studied aspects of clonality is physiological integration between connected ramets. Liu et al. review impacts of clonal integration on tolerance of stress, invasiveness, competitive interactions, community structure, and productivity. One gap in knowledge of clonal integration has been a lack of studies on epiphytic clonal plants. Lu et al. report that clonal integration increased the performance of two rhizomatous, epiphytic ferns in a subtropical, montane, moist forest in China. One next step in the study of clonal integration is to understand how effects scale up to the level of the population. Wang, Y.-J. et al. compare effects of positively and negatively correlated patchiness of resources on intraspecific interactions in the rhizomatous herb *Iris japonica* to show that both the arrangement and the scale of patches can significantly affect competitive intensity.

Mechanisms and consequences of synchronous flowering in clonal bamboos are of great basic and practical interest. Wang, W. et al. present data on stand structure, seed bank dynamics, seedling regeneration, culm characteristics, and energy allocation in patches of an arrow bamboo, *Fargesia qinlingensis*, in patches that did and did not flower during five years after a mass flowering event. Results suggest that habitat modification was most likely responsible for the delayed flowering.

The interaction between clonality and invasion has received considerable attention, and interaction between clonal growth and climate change has begun to attract interest. Researchers are just beginning to tackle three-way interaction between clonality, invasion, and climate change. Clonal plants are widely represented in nature and dominate a number of plant communities and ecosystems around the world, evidence of the ecological importance of clonal growth. Understanding interactions that involve clonal growth will help us predict the impacts of ongoing, rapid change at a global scale in natural environments. We hope that publication of this research topic will stimulate more studies on this important nexus in the coming years.

## Author contributions

All authors listed have made substantial and direct intellectual contributions to the work and approved it for publication.

### Conflict of interest statement

The authors declare that the research was conducted in the absence of any commercial or financial relationships that could be construed as a potential conflict of interest.

